# Open questions: respiratory chain supercomplexes—why are they there and what do they do?

**DOI:** 10.1186/s12915-018-0577-5

**Published:** 2018-11-01

**Authors:** Judy Hirst

**Affiliations:** The Medical Research Council Mitochondrial Biology Unit, University of Cambridge, Wellcome Trust/MRC Building, Cambridge Biomedical Campus, Hills Road, Cambridge, CB2 0XY UK

## Abstract

In the mitochondrial inner membrane the respiratory enzymes associate to form supramolecular assemblies known as supercomplexes. The existence of supercomplexes is now widely accepted—but what functional or structural advantages, if any, do they confer?

## Comment

**Fig. 1:**
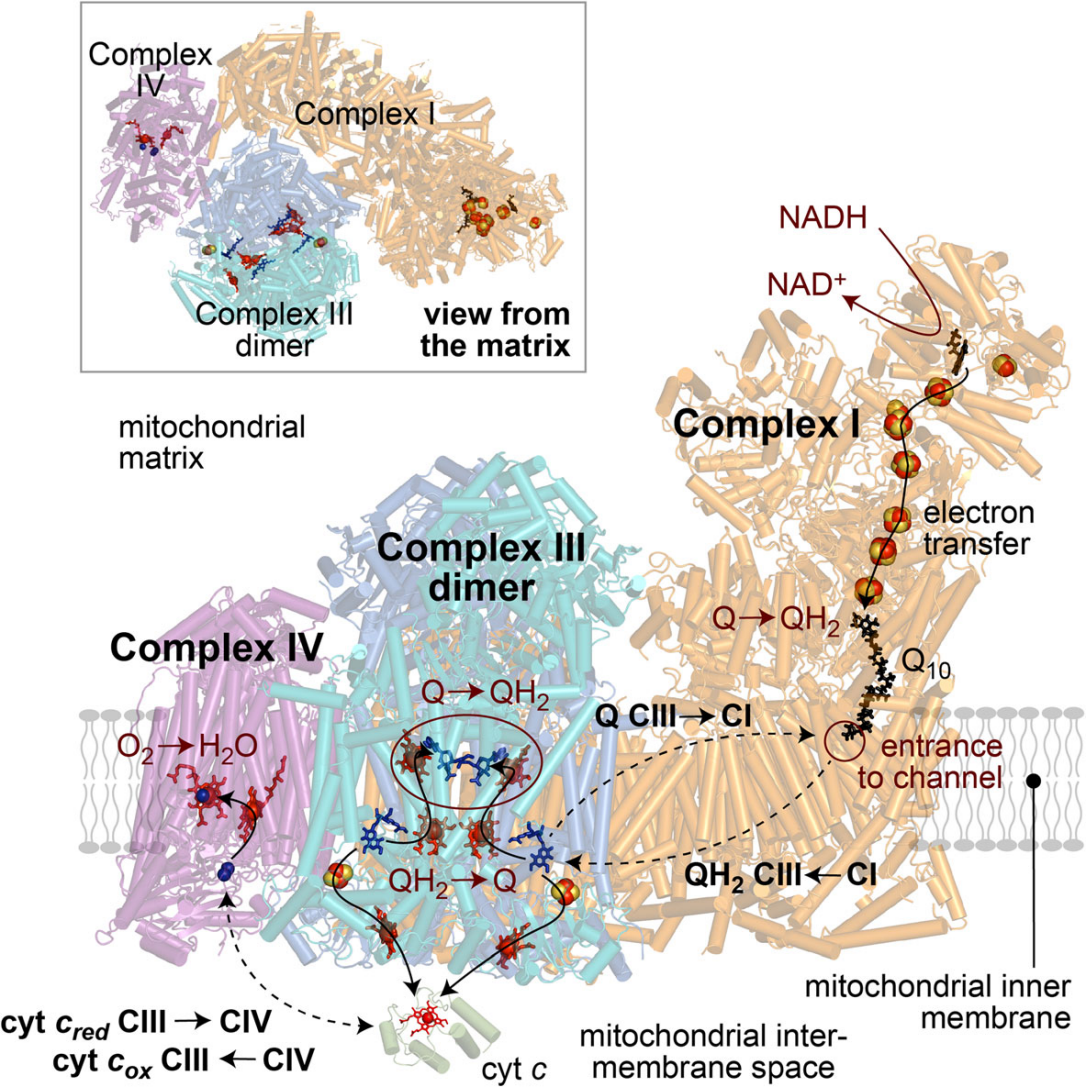
Reactions catalysed by the respirasome supercomplex. The respirasome comprises complexes I, III and IV. Complex I catalyses oxidation of NADH coupled to reduction of ubiquinone-10 to ubiquinol; the entrance to the ubiquinone-binding channel is marked. Complex III (present as a dimer) catalyses reoxidation of ubiquinol, but in a ‘bifurcation’ reactions separates the two electrons that are generated: one is passed to cytochrome *c*, the other is recycled back across the membrane to reduce a further ubiquinone. Each cycle comprises sequential oxidation of two ubiquinol and reduction of one ubiquinone; the recycling increases the proton-pumping stoichiometry. Cytochrome *c* is oxidised by complex IV, where the electrons are consumed in reduction of O_2_ to water. The same structure is shown in the inset viewed from the matrix side of the membrane. Figure created from the structures of the respirasome from porcine heart mitochondria at 5.4 Å resolution (Gu et al., 2016 [3]) and bovine cytochrome *c* (6FF5.PDB), with higher-resolution structures for complex I (6G2J.PDB) and inhibitor-bound complex III (1PPJ.PDB) used to mark the flavin and bound Q/QH_2_

As a major driver of ATP synthesis, the mitochondrial respiratory chain is as central to mammalian metabolism as its dysfunctions are to human disease. In the chain, three major respiratory complexes, complexes I, III and IV, catalyse the step-wise transfer of electrons from NADH (generated from the food we eat) to O_2_ (from the air we breathe). The oxidation of NADH by O_2_ releases a lot of energy, which is trapped by the complexes in transporting protons across the inner mitochondrial membrane, charging it up like a biological battery. The battery is discharged as protons flow back across the membrane through the ATP synthase rotor, turning it to generate ATP. Although the three complexes can each function perfectly well in isolation (complex I oxidises NADH and reduces ubiquinone-10 (Q) to ubiquinol-10 (QH_2_); complex III reoxidizes the QH_2_ (back to Q) and reduces cytochrome *c*; complex IV reoxidizes the cytochrome *c* and reduces O_2_) it is now well established that, in mitochondria, they are organized into weakly associated supramolecular assemblies known as supercomplexes [1]. The best known supercomplex is the respirasome (Fig. 1); it contains all three complexes, plus the mobile electron carriers Q/QH_2_ and cytochrome *c* that shuttle between the complexes, and is therefore the simplest entity capable of independent respiration. Nevertheless, the complexes do not need to associate into supercomplexes in order to catalyse effectively: the reason (or reasons) why they do so remains enigmatic.

Because supercomplexes are so prevalent, it is easy to assume that they must confer a structural or functional advantage on the respiratory system. A widely held view is that they enhance catalysis by channelling the mobile intermediate substrates from complex to complex—but there is little robust evidence to support this assertion. Recent developments in single-particle electron cryo-microscopy (cryo-EM) have supported a proliferation of structural knowledge on the mammalian respirasome and defined its architecture: the membrane arm of complex I curves around the complex III dimer, and complex IV is located between complexes I and III, on the toe of complex I [2, 3] (Fig. 1). The same core architecture has also been observed in tomographic studies on native membranes, although with variable complements of complex IV in particular [4]. Importantly, it is a long way (~ 100 Å; Fig. 1) from the exit to the complex I Q-binding channel to the closest complex III QH_2_-binding site, and there is no confining protein structure between the two sites to guide diffusion. Similarly, the structures reveal no barriers to the free diffusion of cytochrome *c* between complexes III and IV. Therefore, no physical channels exist and the term ‘substrate channelling’ is inappropriate. In the case of true substrate channelling, typically to prevent a toxic intermediate from escaping into the cellular environment, substrates are contained as they move between sites to prevent them exchanging with substrates outside [5]. Although channels may also decrease the transit time between sites, they represent a higher level of organisation for this purpose than simply placing the sites in close proximity to one another—as occurs in the respirasome, as well as in any tightly packed environment.

The term substrate channelling implies that each supercomplex sequesters and retains its own individual complement of Q/QH_2_ and cytochrome *c*. Elegant spectroscopic measurements were used to demonstrate that cytochrome *c* in *Saccharomyces cerevisiae* does not encounter major barriers to free diffusion between complexes or supercomplexes [6] and, in 2014, we reported a set of biophysical experiments on submitochondrial particles and membrane preparations from bovine heart mitochondria that also argued against channelling and sequestration [7]. In particular, coupled mammalian mitochondrial membrane preparations support substantial rates of ‘reverse electron transfer’, a reaction in which QH_2_ generated by complex II outside the respirasome is recycled by complex I inside the respirasome. Reverse electron transfer demonstrates how Q/QH_2_ is able to exchange, on a physiologically relevant timescale, in and out of respirasomes. More recently, in a conceptually simple demonstration of Q/QH_2_ exchange, we incorporated an alternative quinol oxidase (AOX) into bovine heart mitochondrial membranes, to introduce a competing pathway for quinol oxidation [8]. AOX substantially increased the rate of NADH oxidation by O_2_, without affecting the membrane integrity, the supercomplexes, or NADH-linked oxidative phosphorylation. Therefore, the QH_2_ from complex I is reoxidized more rapidly by AOX outside the supercomplex than by complex III inside the supercomplex! The results indicate that substrate channelling does not occur, and is not required to support respiration. Indeed, a strict channelling regime, with a tiny pool of Q/QH_2_ and cytochrome *c* trapped inside each supercomplex would prevent redox sensing through the status of the membrane pool and comprise the ability of the system to withstand the dysfunction of individual complexes.

So, if supercomplexes do not enhance catalysis by substrate channelling, do they present any alternative advantages? Although incorporating the individual complexes into supercomplexes has been suggested to minimise reactive oxygen species production, it is difficult to apply structural or mechanistic knowledge to support this proposal. The local environments of the buried enzyme active sites, at which O_2_ reacts to generate superoxide, are structurally defined sites that are distant from the contact points and interfaces between the complexes. Superoxide production is determined by the levels of reactive intermediates present at those sites (determined in turn by the mechanism and environmental parameters such as substrate concentrations) and by the access of O_2_ to the sites. The latter may be hindered by the adjacent complexes in the supercomplex, but similarly by any complex in the vicinity (by the packing density)—and furthermore, the flavin site of complex I (where NADH is oxidised), which is an important site of superoxide production by the respiratory chain, is exposed outside the supercomplex, at the top of the enzyme’s hydrophilic arm (Fig. 1). Supercomplexes have also been proposed to stabilise the individual complexes, or to provide a scaffold for their assembly. Mutations in the individual complexes often affect the levels of complexes and supercomplexes present, but in particular, mutations that cause defects in complexes III and IV often lead to defects in complex I also [9]. The combined defect suggests a communication between the complexes which could be mediated by the supercomplex structure. However, a supercomplex-independent mechanism also exists to explain the effect: deficiencies in the rate of respiratory-chain catalysis cause the NAD^+^/NADH, fumarate/succinate and Q/QH_2_ pools to become more reduced, and reactive oxygen species production from complex I to increase—and complex I is susceptible to oxidative damage. Finally, it is clear that the levels and composition of supercomplexes in mitochondria respond to changes in the metabolic conditions, increased oxidative stress, or the dysfunction or instability of individual complexes, and may, in fact, be viewed as a sensitive marker of the status of the respiratory system. However, whether changes to the complement of supercomplexes are ever the primary effects that are employed in a mechanistically defined manner to regulate respiratory pathways remains to be established.

Instead, I contend that supercomplexes represent a physical adaptation of the respiratory system to its environment. The mitochondrial inner membrane is an extremely protein-dense environment. As loosely associated but closely packed assemblies, supercomplexes offer a favourable way to allow more complexes to be crammed into the membrane, while avoiding the unfavourable and irreversible interactions that may lead to protein aggregation or degradation. Many of the intercomplex interactions within supercomplexes are formed by the supernumerary subunits—subunits that are not required for the primary catalytic function and that have been accumulated onto the mammalian enzymes through evolution. In complex I, the supernumerary subunits form a cage around the membrane domain. Instead of evolving to stabilise interactions to form the supercomplex, the cage may have evolved to protect the core against too close, restrictive interactions, acting as a fender to hold other complexes away. Furthermore, in mammalian mitochondria, the respiratory supercomplexes occupy the planar cristae surface. Cristae remodelling has been reported to disrupt the supercomplexes [10] and the flat, disc-like structure of the respirasome has likely co-evolved with the membrane topology to create the current situation in which the two depend on one another. Finally, by associating complex I with complex III with complex IV, supercomplexes enforce a homogeneous mixture and distribution of the complexes in the membrane. Within the close-packed homogeneous mixture, the distances that Q/QH_2_ and cytochrome *c* must diffuse between enzymes is minimised—but with no constraint on them to react with their specific partners in the same supercomplex. Therefore, supercomplexes may be instrumental for the stability of the densely and homogeneously packed planar cristae membrane—by supporting this membrane environment they promote the efficient and rapid catalysis of respiration.
